# Disease progression of alpha-mannosidosis and impact on patients and carers – A UK natural history survey

**DOI:** 10.1016/j.ymgmr.2019.100480

**Published:** 2019-06-08

**Authors:** Jacqueline Adam, Rachel Malone, Sioned Lloyd, Jennifer Lee, Christian J. Hendriksz, Uma Ramaswami

**Affiliations:** a MPS Commercial, Amersham, UK; b Chiesi Limited, Manchester, UK; c Decision Resources Group, Manchester, UK; d Steve Biko Academic Unit, Paediatrics and Child Health, University of Pretoria, Pretoria, South Africa; e Lysosomal Disorders Unit, Institute of Immunity and Transplantation, Royal Free London NHS Foundation Trust, London, UK

**Keywords:** Alpha-mannosidosis, Lysosomal storage disorder, Haematopoietic stem cell transplantation, Velmanase alfa, Enzyme replacement therapy, Quality of life

## Abstract

**Introduction:**

Alpha-mannosidosis is an ultra-rare lysosomal storage disorder resulting from the deficient activity of lysosomal alpha-mannosidase. Alpha-mannosidosis presents as a highly heterogenous condition with large variations in symptom severity and disease progression rates. Quantitative and qualitative data for alpha-mannosidosis patients and their caregivers provide important insights into their daily experiences.

**Methods:**

A survey of nine alpha-mannosidosis patients was carried out in the UK between August 2017 and January 2018. Patient demographics, health-related quality of life (HRQoL), and qualitative data from patients and carers relating to clinical characteristics and impact of the disease and treatment were analysed.

**Results:**

At the time of survey completion, patient age ranged from 7 to 37 years. Five patients were described as ‘walking unassisted’, one as ‘walking with assistance’, one as ‘wheelchair-dependent’, and two as ‘severely immobile’. In addition to best supportive care, three patients had received haematopoietic stem cell transplantation (HSCT) and one had received velmanase alfa enzyme replacement therapy (ERT). Patient HRQoL results for the EQ-5D-5 L questionnaire and the Health Utilities Index-3 showed that patients with more severe ambulatory health states reported lower utility values than patients who were more mobile. Patients who received HSCT or ERT experienced improved HRQoL. Carer HRQoL results for the Hospital Anxiety and Depression Scale and Caregiver Strain Index demonstrated that carers experience high levels of stress and anxiety from their caregiving responsibilities.

**Conclusions:**

This survey confirmed the heterogeneity of alpha-mannosidosis and the large impact of the disease and treatment on patients, carers, and families. Early diagnosis and access to treatment offers the best chance of slowing the disease progression and may provide some relief to patients and carers.

## 1 Introduction

Alpha-mannosidosis (OMIM 248500) is an ultra-rare lysosomal storage disorder, with a prevalence estimated at 1:250,000 to 1:1,000,000 live births [1,2]. This hereditary and progressive disorder results from the deficient activity of lysosomal alpha-mannosidase (E.C.3.2.1.24) owing to mutations in the *MAN2B1* gene (609458). Alpha-mannosidase deficiency leads to intracellular accumulation of mannose-containing oligosaccharides and consequently impaired cell function and apoptosis.

Alpha-mannosidosis presents as a highly heterogenous condition and is characterised by a broad spectrum of clinical manifestations, including immunodeficiency, hearing impairment, facial and skeletal abnormalities, and cognitive impairments [3]. Disease-modifying treatments for alpha-mannosidosis are limited, with most patients receiving best supportive care, which focusses on addressing symptoms and clinical features as they arise. Beyond best supportive care, treatment options include allogeneic haematopoietic stem cell transplantation (HSCT) [4] and, in Europe as of April 2018, velmanase alfa enzyme replacement therapy (ERT) [5] where it is indicated for the treatment of non-neurological manifestations in patients with mild to moderate alpha mannosidosis.

Given the broad range of symptoms experienced by patients, the large number of different healthcare professionals they are likely to interact with, and the degenerative nature of the disease, alpha- mannosidosis places a large burden on patients and their families. Rare-disease strategies across Europe note the importance of a qualitative understanding of managing and coping with a rare disease and the experiences of families caring for these patients [6,7].

In addition to recording the pathophysiological aspects and natural history of alpha-mannosidosis, a primary aim of this survey was to understand the individual journey experienced by each patient with alpha-mannosidosis and their families, the challenges they face in everyday life, and the impact this has on both patient and carer quality of life (QoL). This paper presents the demographic characteristics of patients with alpha-mannosidosis, outcomes of the validated QoL measures of the patients and their carers, and qualitative data from patients and carers relating to clinical characteristics and impact of the disease and treatment on QoL, health, and social integration, as retrospectively reported by the patients or carers.

## 2 Methods

### 2.1 Participants

The survey was carried out between August 2017 and January 2018. Patients were eligible to participate in the survey if they had a confirmed diagnosis of alpha-mannosidosis, were enrolled in the MPS Society UK's Registry, and were resident in the UK. Broad consent was given by patients and carers at point of enrolment into the MPS Society's Registry to be contacted and invited to participate in surveys and research projects. At initial contact, all patients and carers were sent an information pack, which contained copies of the surveys in addition to the relevant consent/assent forms which were completed by patients or carers. Carers were all identified as family members and survey responses were fully pseudonymised.

### 2.2 Survey design

The survey was conducted in three phases, involving one postal questionnaire (phase one) and two interviews (phases two and three), which were conducted either face-to-face or via teleconference. During each phase, responses were sought from both patients and carers through a patient survey and a carer survey. Although patient-reported data were sought, all carers were also asked to respond as proxy in addition to patient responses to allow comparison of results. Due to the cognitive impairment of patients, patient-collected data was unable to be obtained for all patients, and when collected may have been unreliable, as such proxy results were used as baseline. Carers also completed separate surveys to determine the impact of caring for someone with alpha-mannosidosis on their own QoL. Patient demographics were collected during each phase. Quantitative data included current age, age at diagnosis of alpha-mannosidosis, treatment received, and walking ability (Table 1).

**Table 1:** Walking ability as defined in multi-stage UK clinical expert (*n* = 5) teleconference interviews.

State	Description
Walking unassisted	The patient is able to walk and go upstairs unassisted
Walking with assistance	The patient requires any form of assistance to walk (e.g. help from another person, footwear to support stability, a walking cane, wheelchair for long distances, hand rails etc.)
Wheelchair dependent	The patient is wheelchair-bound but can still operate walking aids/use assistance to traverse short distances. The patient can still transfer themselves without carer support (e.g. the patient can transfer from the wheelchair into bed independently)
Severe immobility	The patient requires a wheelchair/mobility device continuously and cannot transfer independently (i.e. requires hoists and other assistive equipment)

In phase one, quantitative data relating to the natural history of alpha-mannosidosis were collected. In phases two and three, both quantitative and qualitative data relating to the impact of alpha-mannosidosis and treatment on the patients, carers, and families were collected. Phase two also included validated patient QoL questionnaires [EuroQol-5 Dimension-5 Level questionnaire (EQ-5D-5 L) or EuroQol-5 Dimension-Youth questionnaire (EQ-5D-Y), and Health Utility Index–3 (HUI-3)] and carer QoL questionnaires [Hospital Anxiety and Depression Scale (HADS) and Caregiver Strain Index (CSI)]. All questionnaires used are available as supplementary data.

### 2.3 Validated quality-of-life questionnaires

The EQ-5D-5 L and EQ-5D-Y questionnaires are generic standardised measures of health status developed by the EuroQol group that are applicable to a wide range of health conditions and therapies [8]. Each questionnaire also contains an EQ Visual Analogue Scale (VAS) assessing self-rated health on a scale of 1 to 100, with 1 indicating worst health and 100 indicating best health. In order to compare values for 3-level and 5-level value sets, a crosswalk link function was used to map utility values to the 5-level version, based on the existing value sets for the 3-level version. In this survey, patients (self-report or by proxy) completed this questionnaire.

The HUI-3 questionnaire is a generic health status measure that includes a comprehensive health status classification system and overall health-related QoL utility value that is applicable to all people aged ≥ 5 years in both clinical and general populations [9]. It comprises eight attributes: vision, hearing, speech, ambulation, dexterity, emotion, cognition, and pain. There are five to six levels of functional ability/disability within each attribute, with 1 indicating ability and 5 or 6 indicating severe disability. In this survey, patients (self-report or by proxy) completed this questionnaire.

To assess the impact of caring for someone with alpha-mannosidosis on QoL, two questionnaires were completed by carers. The HADS questionnaire was selected by the investigators during the trial as a measure of anxiety and depression in a general clinical population [10] and the CSI questionnaire was selected as a measure of strain related to care provision [11]. The major domains of strain measured by CSI are physical health, time demands, social interactions, employment, and finances. Responses for each item were either positive or negative.

### 2.4 Statistical methods

Patient responses (self- and/or proxy) to the survey questions were analysed. Frequency distributions were used for categorical variables. Analyses for walking ability (ambulatory health state) were performed using simple pooling. Patients with the same walking ability were combined. Descriptive statistics were used to assess pooled data. Responses to open-ended questions were listed.

For the EQ-5D-5 L, EQ-5D-Y, and HUI-3 questionnaires, analyses were performed using algorithms/tariffs as specified in each respective questionnaire manual to translate raw questionnaire responses into utility values [8,9,12]. Utility values for EQ-5D-Y were mapped to EQ-5D-5 L using the nonparametric crosswalk method [13]. For the HADS and CSI questionnaires, responses were calculated and compared against the thresholds as specified in the questionnaire manuals [10,11].

## 3 Results

### 3.1 Patient demographics

At the time of this survey, 24 patients were enrolled in the MPS Society UK Registry and all were invited to take part. Responses for nine unique patients (38%) were received. Patient self-reported data were collected in three cases, and patient data reported by proxy were collected in all nine cases. Patient demographics are summarised in Table 2. At the time of survey completion, patient age ranged from 7 to 37 years. In all cases, symptoms were present from an early age (birth to 5 years). There was a broad range of ages at which alpha-mannosidosis was diagnosed, with the majority (7 of 9, 78%) diagnosed in childhood or adolescence. Four patients (44%) had received disease-modifying treatment for alpha-mannosidosis in addition to best supportive care. Of these patients, three (one paediatric and two adolescents) received allogeneic HSCT – two patients at 3 years of age, one at 5 years. All three patients received allogeneic HSCT within a year of diagnosis with alpha-mannosidosis. One patient received velmanase alfa ERT as part of a clinical trial at age 30, 15 years after diagnosis. At the time of survey completion, this patient remained on compassionate use with ERT.

**Table 2:** Patient demographics and disease characteristics.

Demographic	Patients with AM (*N* = 9)			
Age range^a^, years	7–37			

Age group^a^				
≥ 6–11 years	1			
12–17 years	3			
≥ 18 years	5			

Age at first symptoms				
< 18 years	9			
≥ 18 years	0			

Age at diagnosis^b^				
< 6 years	3			
≥ 6–11 years	1			
12–17 years	2			
≥ 18 years	2			
Range	17 months–23 years			
	≥ 6–11 years	12–17 years	≥ 18 years	All age groups
	(*n* = 1)	(*n* = 3)	(*n* = 5)	(*n*= 9)
Time since diagnosis^a^, years				
Range	2	9–14	2–27^c^	2–27^c^

Treatment for AM				
BSC	0	1	4	5
BSC + allogeneic HSCT^d^	1	2	0	3
BSC + ERT	0	0	1	1

Walking ability^a^, ^b^, no.				
Unassisted	1	2	2	5
With assistance	0	0	1	1
Wheelchair-dependent	0	0	1	1
Severely immobile	0	1	1	2

Abbreviations: AM, alpha-mannosidosis; BSC, best supportive care; ERT, enzyme replacement therapy; HSCT, haematopoietic stem cell transplantation.

a At time of survey completion.

b Responses taken from phase 3 of the survey responses (most recent data), except for one patient with missing data.

c 2-year age range given, upper limit may be as low as 25 years.

d Treatment was described as bone marrow transplant in survey responses.

The walking ability of patients was ascertained as per the definitions outlined in Table 1. Over half of patients (5 of 9, 56%) were described as ‘walking unassisted’. One patient was described as ‘walking with assistance’, one as ‘wheelchair-dependent’, and two as ‘severely immobile’. All three patients (one adolescent and two adults) who were described as ‘wheelchair-dependent’ or ‘severely immobile’ had not received treatment beyond best supportive care for alpha-mannosidosis and had experienced symptoms of alpha-mannosidosis for at least 14 years.

### 3.2 Patient quality-of-life assessments

Of the nine unique patients, three provided self-reported patient utility values and nine were received by proxy via the EQ-5D-5 L or EQ-5D-Y and HUI-3 questionnaires. Responses by proxy are reported here for overall consistency, comparability, and greater contextual information. Discrepancies between patient self-reported (*n* = 3) and respective proxy-reported responses were noted and were attributed to the reduced cognitive ability of these patients, and as a result proxy-reported results were considered more reliable. Carers also provided their own responses to the CSI and HADS questionnaires. Overall, it was seen from the EQ-5D-5 L and HUI-3 utility values that there was a high, yet heterogeneous, disease impact of alpha-mannosidosis on patient health-related quality of life (HRQoL).

**Fig. 1:**
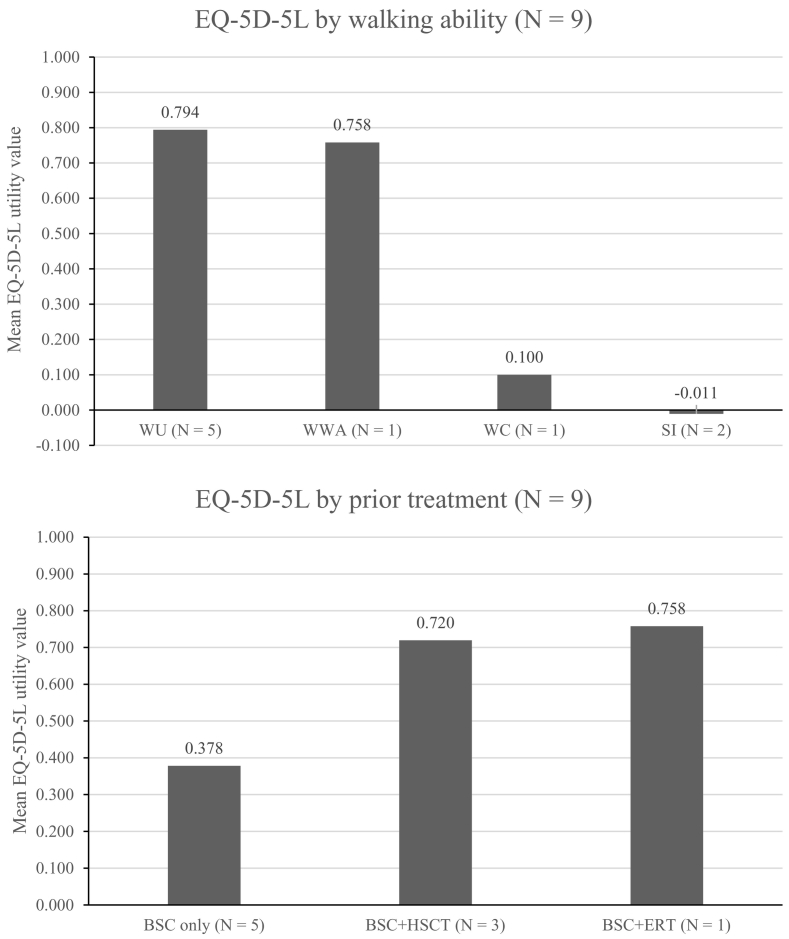
HRQoL of nine patients as reported by proxy measured using the EQ-5D-5 L questionnaire according to walking ability (top) and prior treatment (bottom). A score of 1 indicates ‘perfect health’, a score of 0 indicates ‘death’. Negative values indicate ‘feeling worse than death’. Utility values for two patients were mapped from EQ-5D-Y to EQ-5D-5 L using the nonparametric crosswalk method [15]. Abbreviations: BSC, best supportive care; EQ-5D-5 L, EuroQol 5 Dimensions 5 Levels; ERT, enzyme replacement therapy; HRQoL, health-related quality of life; HSCT, haematopoietic stem cell transplantation; SI, severe immobility; WC, wheelchair-dependent; WU, walking unassisted; WWA, walking with assistance.

**Fig. 2:**
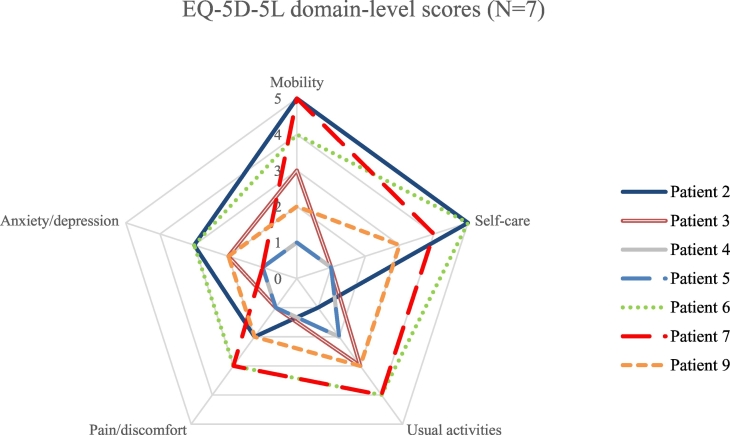
HRQoL by domain of seven patients as reported by proxy measured using the EQ-5D-5 L questionnaire. Each line represents the carer-reported domain score by proxy for an individual patient (*N* = 7). Please note that identical scores were reported for patients 4 and 5 so these appear to be one line. Responses for the EQ-5D-Y questionnaire were completed for two patients; these are not represented here as each dimension on the EQ-5D-Y has three levels compared with five levels on the EQ-5D-5 L. Higher scores indicate a higher level of perceived problems for that domain of the EQ-5D-5 L questionnaire. Abbreviations: EQ-5D-5 L, EuroQol 5 Dimensions 5 Levels; EQ-5D-Y, EuroQol-5 Dimension-Youth; HRQoL, health-related quality of life.

Responses were completed for seven patients for the EQ-5D-5 L questionnaire, and for two patients for the EQ-5D-Y questionnaire who were aged <16 years. Utility values for EQ-5D-Y were mapped to EQ-5D-5 L using the nonparametric crosswalk method [13]. Patients with the least mobility had the lowest EQ-5D-5 L utility values, indicating a lower HRQoL. Patients who were described as severely immobile (*n* = 2) had a mean utility value of −0.011 compared with a mean utility value of 0.794 for patients who were described as walking unassisted (*n* = 5) (Fig. 1). The lower utility values for these patients result from the severely reduced mobility itself and the impact this has on the patients' ability to undertake self-care activities. This result can be seen from the mobility and self-care domain values for these patients (Supplementary Table 1). It must also be noted, however, that reduced mobility is multifactorial and a decline in mobility may be a marker of CNS disease, which also effects self-care. Patients who had no prior treatment for alpha-mannosidosis beyond best supportive care reported poor HRQoL. Most patients in this subgroup (4 of 5, 80%), with an age range of 16 to 37 years, are adults who have lived with this progressive condition for a long period; all three patients who were wheelchair-dependent or severely immobile were in this group. Interestingly, the plot of EQ-5D-5 L scores by domain demonstrated heterogeneity of impact of disease on HRQoL (Fig. 2). There was a wide range of VAS scores for self-rated health across all walking abilities and prior treatment status (mean 74.8, range 33–100). A comparison between patient-reported and proxy-reported responses for the three patients who responded showed that patients tended to report a lower score than carers in the ‘usual activities’ domain.

**Fig. 3:**
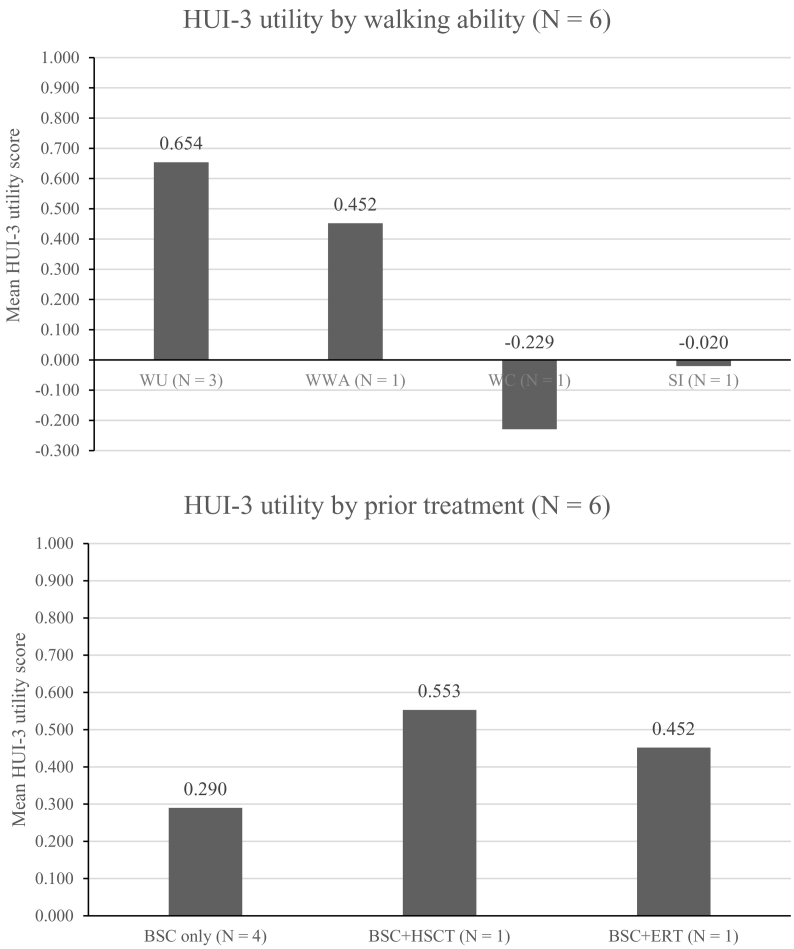
HRQoL of six patients as reported by proxy measured using the HUI-3 questionnaire by walking ability (top) and prior treatment (bottom). HUI-3 utility scores could be calculated for six patients; no data were available for the other three patients. Utility scores are calculated by applying a formula that attaches weights to each of the levels in each dimension. A utility score of 1 indicates ‘perfect health’, a value of 0 indicates ‘death’. Negative values indicate ‘feeling worse than death’. Abbreviations: BSC, best supportive care; ERT, enzyme replacement therapy; HUI-3, Health Utility Index–3; HRQoL, health-related quality of life; HSCT, haematopoietic stem cell transplantation; SI, severe immobility; WC, wheelchair-dependent; WU, walking unassisted; WWA, walking with assistance.

Six complete HUI-3 questionnaires were obtained by proxy, data for the remaining three patients were incomplete and scores were unable to be calculated. Analysis of the HUI-3 utility scores for the six patients showed that HRQoL was again most negatively affected for patients who were described as wheelchair-dependent or severely immobile and patients who had no prior treatment for alpha-mannosidosis beyond best supportive care (Fig. 3). Notably, cognition was the most severely affected attribute, with a score indicating moderate-to-severe disability recorded in 6 patients (83%). Patients who were wheelchair-dependent or severely immobile also had prominent areas of moderate-to-severe impairment in hearing. When comparing patient-reported and proxy-reported responses, two of the three patients reported no or mild disability for cognition compared with the moderate level reported by proxy. Two of the three patients reported a higher overall HUI-3 utility score than those reported by proxy.

### 3.3 Carer quality-of-life assessments

Responses were received from all nine carers for the HADS and CSI questionnaires to assess carer anxiety and depression, and caregiver strain, respectively. Eight carers also reported how much time they spent caregiving each day. Overall, these measures demonstrated that caring for someone with alpha-mannosidosis has a large impact on carer QoL.

**Fig. 4:**
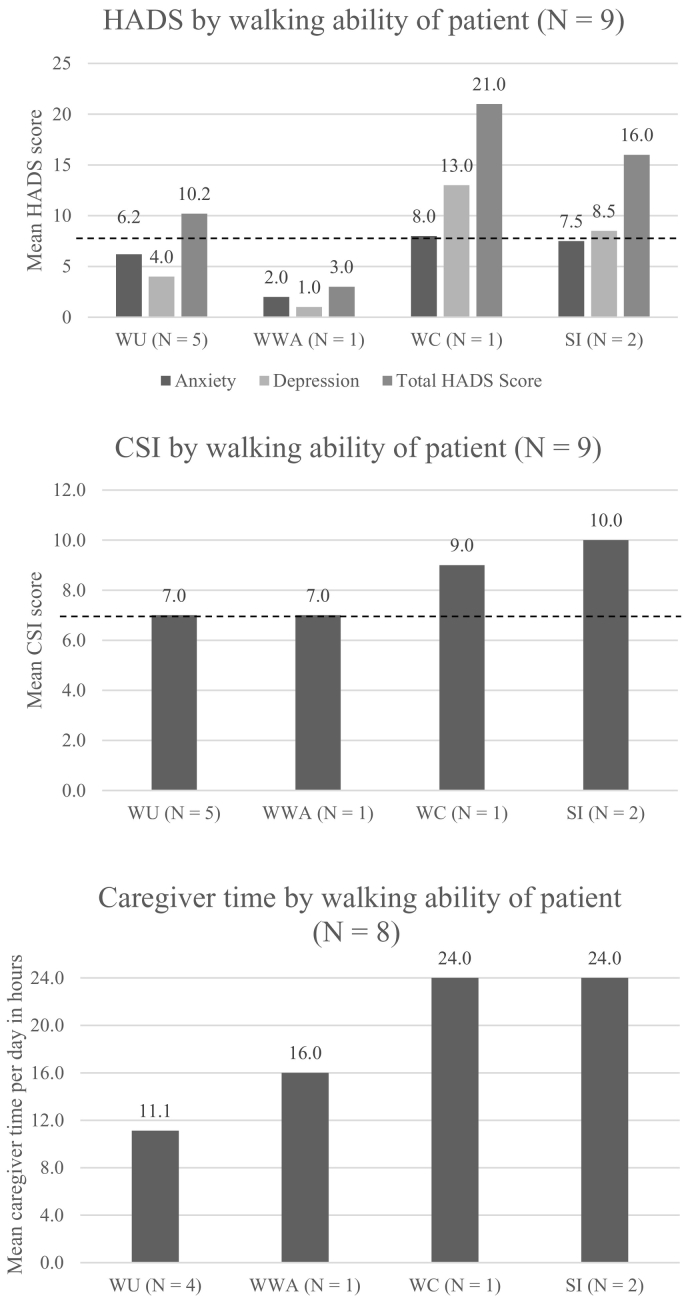
QoL of carers measured by walking ability of the patient using the HADS questionnaire (top), CSI questionnaire (middle) and caregiver time per day (bottom) For HADS, carer scores for anxiety and depression subscales go up to 21 each. 0–7: no case; 8–10: borderline abnormal case; 11–21: abnormal case. For CSI, carer scores go up to 12, with a score of 7 or higher indicating the carer is under a high level of stress related to care provision. Blue horizontal dotted lines indicate the level at which there is a borderline abnormal case for HADS for each subscale or a high level of stress related to care provision for CSI. Abbreviations: CSI, Caregiver Strain Index; HADS, Hospital Anxiety and Depression Scale; HRQoL, health-related quality of life; SI, severe immobility; WC, wheelchair-dependent; WU, walking unassisted; WWA, walking with assistance. (For interpretation of the references to colour in this figure legend, the reader is referred to the web version of this article.)

The HADS anxiety and depression scores are presented in Table 3. Analyses showed that at least half of carers surveyed (5 of 9, 56%) reported having a borderline abnormal or abnormal case in at least one subscale. Whilst a ‘normal’ HADS score is not defined in the literature, experts drawing on experience commonly classify combined scores of 8–10 or 11–21 as borderline abnormal or abnormal cases, respectively. One carer had a borderline abnormal case of both anxiety and depression, and another carer had a borderline abnormal case of anxiety and an abnormal case of depression. QoL was most negatively affected for carers of patients who were described as wheelchair-dependent or severely immobile; however, caring for a patient with less severe ambulatory health states was not necessarily associated with decreased carer anxiety or depression (Fig. 4).

**Table 3:** HADS scores for nine carers of patients with alpha-mannosidosis. For HADS, carer scores for anxiety and depression subscales go up to 21 each. 0–7: no case; 8–10: borderline abnormal case; 11–21: abnormal case. Abbreviation: HADS, Hospital Anxiety and Depression Scale.

HADS subscale	Mean (range)	Normal case	Borderline abnormal case	Abnormal case
		n (%)	n (%)	n (%)
Anxiety	6.2 (2−10)	5 (56%)	4 (44%)	0
Depression	5.7 (0−13)	6 (67%)	2 (22%)	1 (11%)

For the CSI questionnaire, carers had a mean score of 7.9 (range 5–11) for the level of stress related to care provision. A score of 7 or more, indicating high levels of stress related to care provision, was seen in seven of nine carers (78%). QoL was most negatively affected for carers of patients who were wheelchair-dependent or severely immobile (Fig. 4).

The mean time spent caregiving per day was 16.6 h (range 4–24). The number of hours spent per day caring for the patient increased as the ambulatory health state of the patient deteriorated (Fig. 4).

### 3.4 Qualitative analyses

All patient-reported and proxy-reported qualitative responses were analysed to assess the natural history of alpha-mannosidosis, the pathway to diagnosis, and the impact of the disease and treatment on the patients, carers, and their families. It was noted that initial signs and symptoms of alpha-mannosidosis were present from an early age (birth to 5 years) for all patients, with patients and/or carers reporting at least one of the following initial symptoms: facial features, umbilical hernia, talipes (club foot), enlarged head, spine curvature, hearing impairment, dislocated hips, hepatosplenomegaly (enlarged liver and spleen), cognitive impairment, and respiratory, ear, and eye infections. The age at diagnosis varied widely (17 months to 23 years), though seven of nine (78%) were diagnosed in childhood or adolescence (< 18 years).

The majority of patients were diagnosed by a metabolic specialist; however, many were seen by a range of healthcare professionals and specialists before receiving a diagnosis of alpha-mannosidosis. A typical pathway to diagnosis involved GP appointments followed by referrals to various specialists before eventual diagnosis by metabolic specialists. The number of GP appointments prior to specialist referral varied from a single visit to >25 visits; for one patient, a health visitor was seen more often than the GP. The number of GP visits were retrospectively reported, and thus patient recalls may not be accurate. Initial symptoms that prompted the first GP visit included curved spine, not meeting milestones, enlarged head, and hearing problems. Referral to a specialist occurred between birth to over 5 years of age with a median age at referral of 18 months. Most patients received their first specialist referral more than a year and up to 5 years after first presenting with symptoms. For over half of the patient cases in the survey, at least three specialist referrals were required before receiving a formal diagnosis. One patient was first referred to a specialist within months of a GP visit with initial symptoms of delayed walking and talking and an abnormally large head, but the speed of this referral was unusual and may have been due to the symptom presentation and proximity to a centre of excellence in lysosomal storage disorders.

Following diagnosis, patients accessed a broad range of healthcare services, including physiotherapy and occupational therapy, speech and language therapy, and wellbeing counselling. All patients received speech and language therapy during their schooling years; notably, two of these patients only received their diagnosis of alpha-mannosidosis in adulthood. Social services accessed included Disability Living Allowance (DLA), Personal Independence Payment (PIP), Employment Support Allowance (ESA), and Jobseeker's Allowance (JSA). Two patients also received support in the form of an education, health, and care (EHC) plan for special education needs.

The clinical manifestations of alpha-mannosidosis have a considerable impact on patient health and HRQoL. In addition to the initial symptoms as previously stated, all patients reported hearing problems, including deafness (2 of 9, 22%), hearing impairment (2 of 9, 22%), glue ear (fluid build-up in the ear canal; 4 of 9, 44%), and ear infections (1 of 9, 11%). As a result, eight received hearing aids (2 of 9, 22%) or grommets (6 of 9, 67%). Sleep apnoea was reported for two patients (22%), one of whom required a BiPAP (biphasic positive airways pressure) machine. Many patients underwent surgical procedures to treat the symptoms and physical manifestations of alpha-mannosidosis, such as surgery on bones and/or joints (5 of 9, 56%), and removal of adenoids and/or tonsils (4 of 9, 44%). All clinical events mentioned above generally first emerged in childhood or adolescence.

Of the five adult patients surveyed, two were employed part time and one was at college three days a week. All three were described as walking unassisted or walking with assistance. Employment data for the other two adults were missing.

The impact of caring for someone with alpha-mannosidosis is evident from carer responses. Patients' carers and families reported considerable pressure involved in caring for a family member with alpha-mannosidosis; the stress and anxiety caused by the disease put pressure on family relationships and dynamics. Alpha-mannosidosis also impaired the social integration of patients, their carers, and their families. Carers reported providing 4–24 h of care per day to patients and, as a result of their caring commitments, most carers (7 of 9, 78%) were unable to work full-time.

Treatment of alpha-mannosidosis beyond best supportive care had an overall positive impact on patients, their carers, and their families. Three patients included in the survey had previously received treatment with allogeneic HSCT and one patient had initiated and continued to receive treatment with velmanase alfa ERT. All patients who received allogeneic HSCT were under 6 years of age at the time of transplantation, with two patients having received allogeneic HSCT at 3 years and one patient at 5 years of age. At the end of the study period, these patients were 12 and 16, and 7, respectively. The carers of all patients who had received allogeneic HSCT noted the value of this treatment. Allogeneic HSCT was reported by carers to improve the patient's alpha-mannosidosis-related symptoms and increase the health and HRQoL of patients and carers. Improvements were seen in patients' physical symptoms, including coordination, infection rates, speech, hearing, and walking. Following allogeneic HSCT treatment, carers commented on a reduction in their anxiety levels, and the ability to better appreciate life and see the bigger picture. All three patients who received allogeneic HSCT were walking unassisted at the time of the survey (average age: 12 years) and had relatively higher mean EQ-5D-5 L utility values of 0.720 compared with 0.378 for patients who received best supportive care only (average age: 20 years) (Fig. 1). The patient who received ERT was 30 years old when treatment was initiated and at the end of the study period was 34 years old. Treatment with ERT was reported by the patient to improve the physical symptoms, causing a reduction in joint pain and rate of ear infections, and a patient-perceived improvement in gait. The health and HRQoL of both the patient and their carers improved. The patient was walking with assistance (using supportive footwear) at the time of the survey. As seen in Fig. 1, the EQ-5D-5 L utility value for this patient was 0.758 compared with 0.378 for patients who received best supportive care only.

## 4 Discussion

The natural history, quality-of-life outcomes, and qualitative data reported in this UK survey support the observation that alpha-mannosidosis is a heterogeneous disease that follows a degenerative course. Clinical manifestations reported in this survey, such as hearing loss and musculoskeletal abnormalities, are in line with output from a longitudinal, prospective study of 43 patients with alpha-mannosidosis receiving best supportive care [14]. Similarly, high incidence of recurrent infections in the first decade of life, which were reported in a long-running (>20 years), retrospective study of 125 patients with alpha-mannosidosis was a consistent observation in this survey [15].

Disease progression varies from patient to patient. In this survey, some patients were able to walk unassisted for >10 years from the initial appearance of symptoms, while other patients progressed to a wheelchair or became severely immobile within the same time frame. These observations are in keeping with the heterogeneity in clinical progression. Such large variations in disease progression of alpha-mannosidosis have been previously noted. The longitudinal study reported large variations in severity and rates of disease progression, with only slight progression of a few clinical findings within the observational period (24 months) [14].

In all patients who responded to the survey, symptoms were present from early childhood (birth to 5 years). However, diagnosis of alpha-mannosidosis was slow in many cases, taking a decade or longer for almost half of the patients surveyed. There were different routes to diagnosis, which could take months or years as initial symptoms were often non-specific and managed by multiple healthcare professionals, rather than being considered as part of a multi-systemic disease. This situation caused frustration to carers and resulted in a delay in diagnosis. Increased awareness of alpha-mannosidosis may lead to shorter time to diagnosis; it was found in this survey that the GPs and specialists (community paediatricians, neurologists, etc.) who referred the patients to metabolic specialists early in the diagnostic pathway played a key role in faster diagnosis. Data from this survey suggest that earlier diagnosis and access to treatment for alpha-mannosidosis beyond best supportive care may improve not only symptoms of the disease, but also quality of life of the patient.

Allogeneic HSCT is typically considered for patients with a severe form of alpha-mannosidosis in patients diagnosed in early childhood [4]. This treatment option has variable outcomes and carries significant morbidity and mortality risks, which increase with the age of the patient [3,16]. Historically, the risk-benefit profile of allogeneic HSCT is more favourable in younger patients; when carried out in young patients, it can slow disease progression, and the risk of transplant-related complications is minimised [3]. Although specific mortality rates for alpha-mannosidosis related to allogeneic HSCT are not available, studies of Hurler's syndrome demonstrate that post-HSCT mortality rates have improved and may continue to improve as experience in allogeneic HSCT increases, techniques are optimised, and procedures evolve, such as therapeutic monitoring for busulfan and better human leukocyte antigen (HLA) matching [17,18].

In one retrospective analysis of 17 patients with alpha-mannosidosis before and after HSCT, the survival rate was 88% [16]. Most patients received allogeneic HSCT before the age of 6 years (median age 3.6 years; range 1.3–23.1 years). Median follow-up post-transplantation was 5.5 years (range 2.1–12.6 years). Overall, all patients showed developmental improvements after HSCT in terms of improved hearing ability in some patients, and mild or progressive dysostosis (bone development); however, no patients achieved normal development [16]. Additionally, positive outcomes with allogeneic HSCT have been reported in a case report of two siblings with alpha-mannosidosis in the UK, who were aged 6 months and 13 years respectively at the time of transplantation [19]. Three patients who responded to this survey had received allogeneic HSCT. In all cases, carers reported that this treatment had improved patients' symptoms, including co-ordination and hearing and reduced rates of infection without receiving long term antibiotic treatments.

Since April 2018, velmanase alfa has been licensed as ERT for the treatment of non-neurological manifestations in patients with mild to moderate alpha-mannosidosis [5]. One patient in this survey had initiated velmanase alfa ERT as part of a clinical trial and continued to receive it through a compassionate use programme. This patient reported several improvements in the physical symptoms of alpha-mannosidosis following ERT including a reduction in joint pain, fewer ear infections, and a patient-perceived improvement in gait. A prospective study of long-term efficacy and safety of velmanase alfa ERT analysed data from 33 alpha-mannosidosis patients. After an average of 29.3 months of treatment exposure, data showed significant improvements in serum oligosaccharide levels and 3-min stair climb tests [20]. Interestingly, improvements in the 3-min stair climb test were even more pronounced in the paediatric subgroup (*n* = 19) compared to adults. Improvements in endurance, pulmonary function, and motor proficiency were also noted up to 48 months [20]. Shortly following the publication of these results, a post-hoc analysis reported the global treatment response for velmanase alfa in alpha-mannosidosis patients (*n* = 15). After 12 months, a global treatment response was achieved by 87% of the velmanase alfa-treated patients [21]. At last observation, data showed 88% of patients achieved global response, including 100% of paediatric patients [21]. These types of improvement are particularly pertinent in the context of a progressively worsening disease that effects the QoL of the patient and their caregivers.

The results of this current survey show that alpha-mannosidosis has a considerable impact on the QoL of patients, their carers, and their families, with patient walking ability as a key factor. Patients with the most severe ambulatory health states had the lowest EQ-5D-5 L utility values, with a mean utility value of −0.011 (range − 0.048–0.027) for two patients who were severely immobile, and 0.100 for one patient who was wheelchair-dependent compared with 0.794 (range 0.592–1.000) for five patients who were described as walking unassisted. Lower scores indicate lower patient HRQoL, and negative utility values show that the ambulatory health states of severely immobile patients are considered worse than death. However, it is acknowledged that grouping patients according to ambulatory health states may have resulted in bias as two of the five patients in the walking assisted group were considered to have a less severe form of alpha-mannosidosis whilst the other three patients in this group had received allogeneic HSCT. The increased dependence of patients who were wheelchair-dependent or severely immobile on their carer for all aspects of daily life results in a higher level of strain related to care provision for the carer and poorer QoL outcomes for the patient. It was noted that in some cases, cognitive impairment meant that patients did not understand the full impact of alpha-mannosidosis on their lives and this may have contributed to the variation in results observed between patient-reported and proxy-reported HRQoL results. All these factors contribute to the greater negative impact on patient and carer QoL as patients progress to more severe ambulatory health states.

Of the three patients who were able to respond on their own, one described the impact of the disease on their health including poor hearing and vision, reduced mobility (due to joint and bone problems), learning difficulties, and difficulties in comprehension and understanding. In contrast, another patient stated that alpha-mannosidosis had not had an impact on their health or QoL, although the carer remarked that the disease had affected the patient's intellectual ability, understanding, and fine motor skills.

Caring for a child or patient with alpha-mannosidosis had a negative impact on the health and QoL of carers, particularly with regards to their mental health. The responsibilities of looking after a child with alpha-mannosidosis caused stress and anxiety for carers. While carer QoL was most negatively affected for carers of patients who were wheelchair-dependent or severely immobile, interestingly, caring for more mobile patients was not necessarily associated with lower carer anxiety or depression.

To try to determine the level of impact of alpha-mannosidosis on patient QoL, this survey used the EQ-5D-5 L questionnaire. This questionnaire has been used to evaluate patient QoL in many other disease areas, allowing comparisons of the utility values for alpha-mannosidosis with those generated for other, more prevalent, conditions. The mean EQ-5D-5 L utility values of patients with alpha-mannosidosis who were walking unassisted (0.794) or walking with assistance (0.758) in this survey were comparable to that reported for patients with moderate rheumatoid arthritis (0.730) [22]. It should be noted that two patients included in this survey who were walking unassisted had presented primarily with mild ataxia-related symptoms. Consequently, the utility values, and self-care and pain/discomfort domain scores in the EQ-5D-5 L for this group of patients are likely to under-represent the level of disability experienced by patients with alpha-mannosidosis. The utility values of patients who were described as wheelchair-dependent (0.100) or severely immobile (−0.011) were much lower than those reported for patients with severe rheumatoid arthritis (0.300) [22], and only slightly higher than those of patients with multiple sclerosis who were bed ridden or completely immobile (−0.049) [23,24]. Patient QoL data were also reported in the longitudinal study [14], and in the velmanase alfa clinical trial analyses [25,26]. All studies showed that patients with alpha-mannosidosis were dependent upon third party assistance in daily living.

Data in this survey provide additional insights into what patients with this ultra-rare, progressive disease and their carers experience daily. Although the number of respondents to this survey was small, representing 40% of the known UK MPS Society's enrolled alpha-mannosidosis population, the quantitative and qualitative data clearly demonstrate the large impact of alpha-mannosidosis on the patient and their families and caregivers. Nonetheless, the small number of respondents constitutes a limitation of this study, and it remains to be elucidated as to whether the results are generalisable to a larger patient population hence results should be interpreted with caution. However, it should be noted that the available patient population for a condition with a low prevalence, such as alpha-mannosidosis, is small and significant efforts were made to include a larger number of patients in this study. Nevertheless, this may introduce a level of bias into the survey results as factors preventing participation have not been captured and thus results may not be generalisable to the entire UK alpha-mannosidosis population. It must also be noted that although this paper sought to understand how the condition progresses and its impact on patients and carer HRQoL, the reliance on patient and carer memory and recall does not allow for accurate measurement of symptom or HRQoL progression over time or as a result of specific treatments.

## 5 Conclusions

Alpha-mannosidosis is an ultra-rare disease. The heterogeneity of this disease means that patients can present symptoms with different severity and progress at different rates. Clinicians should be aware of the red flags of alpha-mannosidosis symptom presentation that may hasten diagnosis and therefore maximise the opportunity for intervention at the earliest stage possible. However, if severely progressive forms of alpha-mannosidosis are identified or for those with severe cognitive impairment, it is unlikely that current treatment options will be effective. This survey demonstrated that patient QoL utility values decreased as the patient's ambulatory health state progressed. Early identification of patients who can benefit from treatment beyond best supportive care and early access to treatment offers the best chance of slowing disease progression and may provide some relief to patients and carers, as evidenced by the differences in QoL scores reported by treated and untreated patients. Even during best supportive care, there is a need to manage symptoms effectively through co-ordination and organisation of care across different medical specialties and treatment centres. Carers of patients with alpha-mannosidosis experience high levels of stress and anxiety from the day-to-day responsibilities they face. Consequently, efforts to better manage and co-ordinate the patient journey could help reduce carer burden and provide the best care for patients. Ultimately, best practice for supporting patients and families coping with a rare disease can only be formulated with a clear and rounded understanding of the unique daily challenges they face. Surveys that collate both quantitative and qualitative data for patients and their caregivers are an important source of this information.

## Competing interests

JA is employed by MPS Commercial, Amersham, UK. MPS Commercial is a wholly owned, not-for-profit subsidiary of the Society for Mucopolysaccharide Diseases (MPS Society) which is a registered charity. Our social objectives are to invest any profits into the MPS community for the purposes of education, enhancing needs-led advocacy support, quality-of-life research and scientific research. Chiesi paid MPS Commercial to carry out this study. MPS Commercial also receive grants and are paid for research into rare diseases (other than alpha-mannosidosis), by the following Pharma: Shire, BioMarin, Amicus, Ultragenyx, Sanofi, Lysogene, Sobi, Orchard and Alexion.

RM and SL are employed by Chiesi Limited, Manchester, UK.

JL has no competing interests to declare.

CJH is CEO and owner of FYMCA Medical Ltd.

UR has received honoraria for lectures and advisory boards from Amicus, Chiesi, Genzyme and Shire.

## Funding sources

This survey was sponsored by Chiesi Limited.

## Ethics approval

Ethics approval by a Research Ethics Committee was not deemed to be necessary for this survey following consultation with the NHS Health Research Authority (HRA) decision tool and subsequent direct discussions with the HRA.

## Appendix A Supplementary data

EQ-5D-5L domains walking ability results Image 1

Patient questionnaires Image 2

## References

[1] Poupětová H., Ledvinová J., Berná L., Dvořáková L., Kožich V., Elleder M. (2010). The birth prevalence of lysosomal storage disorders in the Czech Republic: comparison with data in different populations. J. Inherit. Metab. Dis..

[2] Meikle P.J., Hopwood J.J., Clague A.E., Carey W.F. (1999). Prevalence of lysosomal storage disorders. JAMA..

[3] Malm D., Nilssen Ø. (2008). Alpha-mannosidosis. Orphanet J. Rare Dis..

[4] Prasad V.K., Kurtzberg J. (2010). Cord blood and bone marrow transplantation in inherited metabolic diseases: scientific basis, current status and future directions. Br. J. Haematol..

[5] Ltd Chiesi (2018). Lamzede Summary of Product Characteristics.

[6] EURORDIS, EURORDIS' Position on Rare Disease Research. https://www.eurordis.org/sites/default/files/EURORDIS_Rapport_Research_2012.pdf, 2012 (accessed September 2018).

[7] UK Department of Health and Social Care (2013). UK Strategy for Rare Diseases – Commitments. https://assets.publishing.service.gov.uk/government/uploads/system/uploads/attachment_data/file/285770/rare-disease-commitments.pdf.

[8] EuroQol, EQ-5D-5L User Guide. https://euroqol.org/wp-content/uploads/2016/09/EQ-5D-5L_UserGuide_2015.pdf, 2015 (accessed September 2018).

[9] Furlong W., Feeny D., Torrance G. (2017). Health Utilities Index (HUI®) Procedures Manual. Permission for use of this manual was provided by Health Utilities Inc.

[10] Zigmond A.S., Snaith R.P. (1983). The hospital anxiety and depression scale. Acta Psychiatr. Scand..

[11] Robinson B.C. (1983). Validation of a caregiver strain index. J. Gerontol..

[12] (2015). EuroQol, EQ-5D-Y User Guide. https://euroqol.org/wp-content/uploads/2016/09/EQ-5D-Y_User_Guide_v1.0_2014.pdf.

[13] van Hout B., Jansse M.F., Feng Y.S., Kohlmann T., Busschbach J., Golicki D., Lloyd A., Scalone L., Kind P. (2012). A.S, Pickard, interim scoring for the EQ-5D-5L: mapping the EQ-5D-5L to EQ-5D-3L value sets. Value Health.

[14] Beck M., Olsen K.J., Wraith J.E., Zeman J., Michalski J.C., Saftig P., Fogh J., Malm D. (2013). Natural history of alpha mannosidosis a longitudinal study. Orphanet J. Rare. Dis..

[15] Malm D., Riise Stensland H.M., Edvardsen Ø. (2014). The natural course and complications of alpha-mannosidosis – a retrospective and descriptive study. J. Inherit. Metab. Dis..

[16] Mynarek M., Tolar J., Albert M.H., Escolar M.L., Boelens J.J., Cowan M.J., Finnegan N., Glomstein A., Jacobsohn D.A., Kuhl J.S., Yabe H., Kurtzberg J., Malm D., Orchard P.J., Klein C., Lucke T., Sykora K.W. (2012). Allogeneic hematopoietic SCT for alpha-mannosidosis: an analysis of 17 patients. Bone Marrow Transplant..

[17] Boelens J.J., Wynn R.F., O'Meara A., Veys P., Bertrand Y., Souillet G., Wraith J.E., Fischer A., Cavazzana-Calvo M., Sykora K.W., Sedlacek P., Rovelli A., Uiterwaal C.S.P.M., Wulffraatet N. (2007). Outcomes of hematopoietic stem cell transplantation for Hurler's syndrome in Europe: a risk factor analysis for graft failure. Bone Marrow Transplant..

[18] Boelens J.J., Aldenhoven M., Purtill D., Ruggeri A., Defor T., Wynn R., Wraith E., Cavazzana-Calvo M., Rovelli A., Fischer A., Tolar J., Prasad V.K., Escolar M., Gluckman E., O'Meara A., Orchard P.J., Veys P., Eapen M., Kurtzberg J., Rocha V. (2013). Outcomes of transplantation using various hematopoietic cell sources in children with Hurler syndrome after myeloablative conditioning. Blood..

[19] Broomfield A.A., Chakrapani A., Wraith J.E. (2010). The effects of early and late bone marrow transplantation in siblings with alpha-mannosidosis. Is early haematopoietic cell transplantation the preferred treatment option?. J. Inherit. Metab. Dis..

[20] Lund A.M., Borgwardt L., Cattaneo F., Ardigò D., Geraci S., Gil-Campos M., De Meirleir L., Laroche C., Dolhem P., Cole D., Tylki-Szymanska A., Lopez-Rodriguez M., Guillén-Navarro E., Dali C.I., Héron B., Fogh J., Muschol N., Phillips D., Van den Hout J.M. Hannerieke, Jones S.A., Amraoui Y., Harmatz P., Guffon N. (2018). Comprehensive long-term efficacy and safety of recombinant human alpha-mannosidase (velmanase alfa) treatment in patients with alpha-mannosidosis. J. Inherit. Metab. Dis..

[21] Harmatz P., Cattaneo F., Ardigo D., Geraci S., Hennermann J.B., Guffon N., Lund A., Hendriksz C.J., Borgwardt L. (2018). Enzyme replacement therapy with velmanase alfa (human recombinant alpha-mannosidase): Novel global treatment response model and outcomes in patients with alpha-mannosidosis. Mol. Gen. Mat..

[22] Hernández-Alava M., Pudney S. (2017). Econometric modelling of multiple self-reports of health states: the switch from EQ-5D-3L to EQ-5D-5L in evaluating drug therapies for rheumatoid arthritis. J. Health Econ..

[23] Hendriksz C.J., Lavery C., Coker M., Ucar S.K., Jain M., Bell L., Lampe C. (2014). Burden of disease in patients with Morquio A syndrome: results from an international patient-reported outcomes survey. Orphanet J. Rare Dis..

[24] Orme M., Kerrigan J., Tyas D., Russell N., Nixon R. (2007). The effect of disease, functional status, and relapses on the utility of people with multiple sclerosis in the UK. Value Health.

[25] Borgwardt L., Thuesen A.M., Olsen K.J., Fogh J., Dali C.I., Lund A.M. (2015). Cognitive profile and activities of daily living: 35 patients with alpha-mannosidosis. J. Inherit. Metab. Dis..

[26] Guffon N., Amraoui Y., Cattaneo F., Ardigo D., Geraci S., Gil-Campos M., de Meirleir L., van den Hout J.M.P., Jones S.A., Lund A.M., Borgwardt L. (2017). Improvements in endurance, serum immunoglobulin G levels and quality of life in alpha-mannosidosis patients switching from placebo to velmanase alfa long-term enzyme replacement therapy. J. Inborn Errors Metab. Screen..

