# Supplementary material for: Disease progression of alpha-mannosidosis and impact on patients and carers – A UK natural history survey
Source: Mol Genet Metab Rep. 2019 Jun 8;20:100480. doi: 10.1016/j.ymgmr.2019.100480 (PMC6557729; doi:10.1016/j.ymgmr.2019.100480)
Supplement: Supplementary file 1 — EQ-5D-5L domains walking ability results [file mmc1.docx]

**Supplementary material:** **Mean score for the five EQ-5D-5L domains according to walking ability as defined in multi-stage UK clinical expert (n = 5) teleconference interviews.**Carer-reported data by proxy for patients with alpha-mannosidosis

| **Mean EQ-5D-5L score for domain (coded from 1 to 5)** | **Walking ability** | | | | All patients |
| --- | --- | --- | --- | --- | --- |
|  | **Walking unassisted** | **Walking with assistance** | **Wheelchair dependent** | **Severe immobility** |  |
| **Patients ≥ 16 years, N** | 3 | 1 | 1 | 2 | 7 |
| Mobility | 1.3 | 3.0 | 4.0 | 5.0 | 3.0 |
| Self-care | 1.7 | 1.0 | 5.0 | 4.5 | 2.9 |
| Usual activities | 2.3 | 3.0 | 4.0 | 2.5 | 2.7 |
| Pain/Discomfort | 1.3 | 1.0 | 3.0 | 2.5 | 1.9 |
| Anxiety/Depression | 1.3 | 2.0 | 3.0 | 2.0 | 1.9 |
| **Mean EQ-5D-Y score for domain (coded from 1 to 3)** |  | | | | |
| **Patients < 16 years, N** | 2 | 0 | 0 | 0 | 2 |
| Mobility | 1.5 | - | - | - | 1.5 |
| Self-care | 1.5 | - | - | - | 1.5 |
| Usual activities | 1.5 | - | - | - | 1.5 |
| Pain/Discomfort | 1.5 | - | - | - | 1.5 |
| Anxiety/Depression | 1.5 | - | - | - | 1.5 |

Each dimension for EQ-5D-5L was coded from 1 to 5: no problems, slight problems, moderate problems, severe problems, and unable to function (mobility, self-care and usual activities) or extreme problems (pain/discomfort and anxiety/depression). Each dimension for EQ-5D-Y was coded from 1 to 3: no problems, some problems, a lot of problems.
