# Supplementary material for: Disease progression of alpha-mannosidosis and impact on patients and carers – A UK natural history survey
Source: Mol Genet Metab Rep. 2019 Jun 8;20:100480. doi: 10.1016/j.ymgmr.2019.100480 (PMC6557729; doi:10.1016/j.ymgmr.2019.100480)
Supplement: Supplementary file 2 — Patient questionnaires [file mmc2.pdf]

Supplementary information

Blank surveys (Phase 1–3)

Alpha-Mannosidosis Disease Progression Survey - I - Patient

Patient

1. Patient ID (for office use only)

2. Date of completion

	DD	MM	YYYY
Date	/	/	

3. How old are you (years)?

4. How much do you weigh (kg)?

5. How old were you when you first started experiencing symptoms of alpha-mannosidosis (years)?

6. How old were you when you were diagnosed with alpha-mannosidosis (years)?

7. What is your current walking ability? Please tick which best applies to you.

- ◯ I can walk unaided
- ◯ I can walk with aids (e.g. braces, splints, ankle foot orthoses, crutches, cane, walker)
- ◯ I use a wheelchair
- ◯ I am immobile, need a carer or hoist to transfer, and help from a carer with all daily activities

8. If applicable, how old were you (years), when you first:

Started using walking aids	
Started using a wheelchair	
Needed a carer or hoist to transfer, and help from a carer with all daily activities	

9. How many infections requiring hospital admission have you had in the last year?

[ ]

10. What infections led to you being treated in hospital in the last year? Please indicate how many.

Ear	
Throat	
Chest	
Digestive system	
Other (please specify)	

11. How long did you spend in hospital with an infection? Please indicate the shortest time and the longest time (e.g. number of days, weeks, months).

Shortest time	
Longest time	

12. How many infections not requiring hospital admission have you had in the last year?

[ ]

13. What infections, not requiring hospital admission, have you had in the past year? Please indicate how many.

Ear

Throat

Chest

Digestive system

Other (please specify)

14. How long did these infections last? Please indicate the shortest time and the longest time (e.g. number of days, weeks, months).

Shortest time

Longest time

15. Have you received specific treatment for alpha-mannosidosis (e.g. bone marrow transplant)?

- ○ Yes
- ○ No
- ○ If yes, please specify what treatment and how old you were (years) when you received it.

Please complete this form as fully as you can and return to
MPS Commercial in the stamped addressed envelope provided

Patient Access to Clinical Trials and Treatment

MPS Commercial

Alpha-Mannosidosis Disease Progression Survey - I - Carer

Carer

1. Patient ID (for office use only)

[ ]

2. Date of completion

	DD		MM		YYYY
Date	[ ]	/	[ ]	/	[ ]

3. How old is your child (years)?

[ ]

4. How much does your child weigh (kg)?

[ ]

5. How old was your child when they first started experiencing symptoms of alpha-mannosidosis (years)?

[ ]

6. How old was your child when they were diagnosed with alpha-mannosidosis (years)?

[ ]

7. What is your child's current walking ability? Please tick which best applies to your child.

- ○ They can walk unaided
- ○ They can walk with aids (e.g. braces, splints, ankle foot orthoses, crutches, cane, walker)
- ○ They use a wheelchair
- ○ They are immobile, need a carer or hoist to transfer, and help from a carer with all daily activities

8. If applicable, how old was your child (years), when they first:

Started using walking aids ______

Started using a wheelchair ______

Needed a carer or hoist to transfer, and help from a carer with all daily activities ______

9. How many infections requiring hospital admission has your child had in the last year?

______

10. What infections led to your child being treated in hospital in the last year? Please indicate how many.

Ear ______

Throat ______

Chest ______

Digestive system ______

Other (please specify) ______

11. How long did your child spend in hospital with an infection? Please indicate the shortest time and the longest time (e.g. number of days, weeks, months).

Shortest time ______

Longest time ______

12. How many infections not requiring hospital admission has your child had in the last year?

______

13. What infections, not requiring hospital admission, has your child had in the past year? Please indicate how many.

Ear

Throat

Chest

Digestive system

Other (please specify)

14. How long did these infections last? Please indicate the shortest time and the longest time (e.g. number of days, weeks, months).

Shortest time

Longest time

15. Has your child received specific treatment for alpha-mannosidosis (e.g. bone marrow transplant)?

- ○ Yes
- ○ No
- ○ If yes, please specify what treatment and how old were they (years) when they received it.

Please complete this form as fully as you can and return to
MPS Commercial in the stamped addressed envelope provided

Alpha-Mannosidosis Disease Progression Survey - II - Patient

For patients with alpha-mannosidosis aged 16 years and over

Overview

1. Patient ID (for office use only)

[ ]

2. Date of completion

	DD		MM		YYYY
Date	[ ]	/	[ ]	/	[ ]

3. How old are you (years)?

[ ]

4. How much do you weigh (kg)?

[ ]

5. How old were you when you were diagnosed with alpha-mannosidosis (years)?

[ ]

6. What is your current walking ability? Please tick which best applies to you.

- ○ I can walk unaided
- ○ I can walk with aids (e.g. braces, splints, ankle foot orthoses, crutches, cane, walker)
- ○ I use a wheelchair
- ○ I am immobile, need a carer or hoist to transfer, and help from a carer with all daily activities

Alpha-Mannosidosis Disease Progression Survey - II - Patient

For patients with alpha-mannosidosis aged 16 years and over

Pathway to diagnosis

7. How old were you when you first started to experience symptoms of alpha-mannosidosis (years)?

8. What initial symptoms first prompted you to visit your GP?

9. How many visits to your GP (with these initial symptoms) did you make before you were referred to a hospital doctor (specialist)?

10. Which hospital doctor (specialist) were you first referred to? Tick ONE which applies

- ◯ Ear, nose, throat specialist
- ◯ Bone/joint specialist (Orthopaedic specialist)
- ◯ Eye specialist (Opthalmologist)
- ◯ Lung specialist (Pulmonary specialist)
- ◯ Brain/nerve specialist (Neurologist)
- ◯ Children's doctor (Paediatrician)
- ◯ Alpha-mannosidosis specialist (Metabolic specialist)
- ◯ Other (please specify)

11. How old were you when you were referred to the first hospital doctor (specialist) in years?

12. What happened after your initial referral to a hospital doctor (specialist)?

- ◯ I was treated by the hospital doctor (specialist) - please go to Question 18
- ◯ I was referred to a second hospital doctor (specialist) - please go to Question 13
- ◯ I was treated by the hospital doctor (specialist) and referred to a second hospital doctor (specialist) - please go to Question 13

13. If you were referred to a second hospital doctor (specialist):

How old were you (years)?

Which hospital doctor (specialist) were you referred to?

How many hospital visits did it take for before you were referred to the second hospital doctor (specialist)?

14. What happened after your referral to a second hospital doctor (specialist)?

- ◯ I was treated by the hospital doctor (specialist) - please go to question 18
- ◯ I was referred to third hospital doctor (specialist) - please go to question 15
- ◯ I was treated by the hospital doctor (specialist) and referred to a third hospital doctor (specialist) - please go to Question 15

15. If you were referred to a third hospital doctor (specialist):

How old were you (years)?

[ ]

Which hospital doctor (specialist) were you referred to?

[ ]

How many hospital visits did it take for before you were referred to the third hospital doctor (specialist)?

[ ]

16. What happened after your referral to third hospital doctor (specialist)?

- ◯ I was treated by the hospital doctor (specialist) - please go to question 18
- ◯ I was referred to another hospital doctor (specialist) - please go to question 17
- ◯ I was treated by the hospital doctor (specialist) and referred to another hospital doctor (specialist) - please go to question 17

17. If you were referred to 4 or more hospital doctors (specialists), please list details here:

[ ]

Patient Access to Clinical Trials and Treatment

MPS Commercial

Alpha-Mannosidosis Disease Progression Survey - II - Patient

For patients with alpha-mannosidosis aged 16 years and over

Clinical events and treatment

18. Please describe any important clinical events/illnesses relating to alpha-mannosidosis you have had, how old you were (years) and how long they lasted.

Note to interviewer, please complete for as many clinical events as reported (not just one) and continue on a separate sheet for each event/illness.

Important clinical event/illnesses

Treatment received in response to important clinical event/illness

Age at time of event/illness

Duration of clinical event/illness (e.g.days/weeks/months)

19. Have you ever received treatment(s) for alpha-mannosidosis? (e.g. joint surgery, bone marrow transplant, treatment as part of a clinical trial, cervical compression)

○ Yes - Please go to Question 20

○ No - Please go to Question 23

20. Please provide details of the treatments you have received for alpha-mannosidosis.

Note to interviewer, please complete for as many treatments as reported (not just one) and continue on a separate sheet for each treatment.

Treatment received

Age at when given treatment

Treatment duration (e.g. days/weeks/months)

21. Have any of your alpha-mannosidosis symptoms improved with treatment?

- ○ Yes - Please go to Question 22
- ○ No - Please go to Question 23

22. Please provide details on which symptoms have improved.

Note to interviewer, please complete for as many symptoms as reported (not just one) and continue on a separate sheet for each symptom.

Which symptom (e.g. walking, hearing, infections)

Age at symptom improvement

Duration (e.g. days/weeks/months)

23. How many times do you currently visit hospital each year for alpha-mannosidosis and why?

24. How many times do you currently visit your GP each year for alpha-mannosidosis and why?

Patient Access to Clinical Trials and Treatment

MPS Commercial

Alpha-Mannosidosis Disease Progression Survey - II - Patient

For patients with alpha-mannosidosis aged 16 years and over

Transition to adult services

25. For patients who have transferred from child (paediatric) to adult services, please tell us about the process of transition. What impact did it have? Did you have to move hospital/cities as a result of transitioning to adult services?

Patient Access to Clinical Trials and Treatment

MPS Commercial

Alpha-Mannosidosis Disease Progression Survey - II - Patient

For patients with alpha-mannosidosis aged 16 years and over

Services used

26. What other health services have you accessed for alpha-mannosidosis?

Note to interviewer, for each service start by asking whether the patient has accessed it. If 'Yes' please ask: At what age did you start using the service (years)?; How often do you use this service (per year)?; and Why do you use the service?
If 'No please note this and move to the next service in the list and continue.

Physiotherapy

Occupational therapy

Speech therapy

Wellbeing therapy (e.g. counselling)

Hydrotherapy

Wheelchair/buggy services

Continence services

Prenatal diagnosis services

Other (please specify)

27. What social services have you accessed for alpha-mannosidosis?

Note to interviewer, for each service start by asking whether the patient has accessed it. If 'Yes' please ask: At what age did you start using the service (years)?; How often do you use this service (per year)?; and Why do you use the service?
If 'No please note this and move to the next service in the list and continue.

Housing	
Benefits advice	
Direct payment	
Respite short breaks	
Educational healthcare plans	
Carer's assessment	
Other (please specify)	

Your home

28. Has your home required adaptations to accommodate any disability caused by alpha-mannosidosis? If so, please describe how? What age were you (years) when the adaptations were made to your home?

29. How were adaptations funded?

- ◯ I funded them myself/my family funded them
- ◯ NHS/Social care funding
- ◯ Council disability facilities grant
- ◯ Housing association grant
- ◯ Part external funding and part personal funding

Alpha-Mannosidosis Disease Progression Survey - II - Patient

For patients with alpha-mannosidosis aged 16 years and over

Expenses

30. Have you incurred any personal/out of pocket expenses caused by alpha-mannosidosis? If so please estimate the cost per year, at what age (years) and what was the reason for the expense.

Patient Access to Clinical Trials and Treatment

MPS Commercial

Alpha-Mannosidosis Disease Progression Survey - II - Patient

For patients with alpha-mannosidosis aged 16 years and over

31. Quality of life questionnaires to be administered by interviewer. Patient to respond. Please tick when completed.

- ◯ HUI-3
- ◯ EQ-5D-5L

Patient Access to Clinical Trials and Treatment

MPS Commercial

Alpha-Mannosidosis Disease Progression Survey - II - Carer

For carers of patients with alpha-mannosidosis

Overview

1. Patient ID (for office use only)

2. Date of completion

Date DD / MM / YYYY

3. How old is your child with alpha-mannosidosis (years)?

4. How much does your child with alpha-mannosidosis weigh (kg)?

5. How old was your child when they were diagnosed with alpha-mannosidosis (years)?

6. What is your child's current walking ability? Please tick which best applies to them.

- ◯ They can walk unaided
- ◯ They can walk with aids (e.g. braces, splints, ankle foot orthoses, crutches, cane, walker)
- ◯ They use a wheelchair
- ◯ They are immobile, need a carer or hoist to transfer, and help from a carer with all daily activities

7. How many hours a day (24 hours) do you provide care for your child with alpha-mannosidosis?

Alpha-Mannosidosis Disease Progression Survey - II - Carer

For carers of patients with alpha-mannosidosis

Pathway to diagnosis

8. How old was your child when they first started to experience symptoms of alpha-mannosidosis (years)?

9. What initial symptoms first prompted your child to visit their GP?

10. How many visits to their GP (with these initial symptoms) did your child make before they were referred to a hospital doctor (specialist)?

11. Which hospital doctor (specialist) was your child first referred to? Tick ONE which applies

- ◯ Ear, nose, throat specialist
- ◯ Bone/joint specialist (Orthopaedic specialist)
- ◯ Eye specialist (Opthalmologist)
- ◯ Lung specialist (Pulmonary specialist)
- ◯ Brain/nerve specialist (Neurologist)
- ◯ Children's doctor (Paediatrician)
- ◯ Alpha-mannosidosis specialist (Metabolic specialist)
- ◯ Other (please specify)

12. How old was your child when they were referred to the first hospital doctor (specialist) in years?

13. What happened after their initial referral to a hospital doctor (specialist)?

- ◯ They were treated by the hospital doctor (specialist) - please go to Question 19
- ◯ They were referred to a second hospital doctor (specialist) - please go to Question 14
- ◯ They were treated by the hospital doctor (specialist) and referred to a second hospital doctor (specialist) - please go to Question 14

14. If they were referred to a second hospital doctor (specialist):

How old were they (years)?

Which hospital doctor (specialist) were they referred to?

How many hospital visits did it take for before they were referred to the second hospital doctor (specialist)?

15. What happened after their referral to a second hospital doctor (specialist)?

- ◯ They were treated by the hospital doctor (specialist) - please go to question 19
- ◯ They were referred to third hospital doctor (specialist) - please go to question 16
- ◯ They were treated by the hospital doctor (specialist) and referred to a third hospital doctor (specialist) - please go to Question 16

16. If they were referred to a third hospital doctor (specialist):

How old were they (years)?

Which hospital doctor (specialist) were they referred to?

How many hospital visits did it take for before they were referred to the third hospital doctor (specialist)?

17. What happened after their referral to the third hospital doctor (specialist)?

- ◯ They were treated by the hospital doctor (specialist) - please go to question 19
- ◯ They were referred to another hospital doctor (specialist) - please go to question 18
- ◯ They were treated by the hospital doctor (specialist) and referred to another hospital doctor (specialist) - please go to question 18

18. If your child was referred to 4 or more hospital doctors (specialists), please list details here:

Clinical events and treatment

19. Please describe any important clinical events/illnesses relating to alpha-mannosidosis that your child has had, how old they were (years) and how long they lasted

Note to interviewer, please complete for as many clinical events as reported (not just one) and continue on a separate sheet for each event/illness.

Important clinical event/illnesses

[ ]

Treatment received in response to important clinical event/illness

[ ]

Age at time of event/illness

[ ]

Duration of clinical event/illness (e.g.days/weeks/months)

[ ]

20. Has you child ever received treatment(s) for alpha-mannosidosis? (e.g. joint surgery, bone marrow transplant, treatment as part of a clinical trial, cervical compression)

- ◯ Yes - please go to Question 21
- ◯ No - please go to Question 24

21. Please provide details of the treatment(s) your child has received for alpha-mannosidosis.

Note to interviewer, please complete for as many treatments as reported (not just one) and continue on a separate sheet for each treatment.

Treatment received

Age at when given treatment

Treatment duration (e.g. days/weeks/months)

22. Have any of your child's alpha-mannosidosis symptoms improved with treatment?

- ○ Yes - Please go to Question 23
- ○ No - Please go to Question 24

23. Please provide details on which symptoms have improved.

Note to interviewer, please complete for as many symptoms as reported (not just one) and continue on a separate sheet for each symptom.

Which symptom (e.g. walking, hearing, infections)

Age at symptom improvement

Duration (e.g. days/weeks/months)

24. How many times does your child currently visit hospital each year for alpha-mannosidosis and why?

25. How many times does your child currently visit your GP each year for alpha-mannosidosis and why?

Patient Access to Clinical Trials and Treatment

MPS Commercial

Alpha-Mannosidosis Disease Progression Survey - II - Carer

For carers of patients with alpha-mannosidosis

Transition to adult services

26. If your child has transferred from child (paediatric) to adult services, please tell us about the process of transition. What impact did it have? Did they have to move hospital/cities as a result of transitioning to adult services?

Patient Access to Clinical Trials and Treatment

MPS Commercial

Alpha-Mannosidosis Disease Progression Survey - II - Carer

For carers of patients with alpha-mannosidosis

Services used

27. What other health services has your child accessed for alpha-mannosidosis?

Note to interviewer, for each service start by asking whether their child has accessed it. If 'Yes' please ask: At what age did they start using the service (years)?; How often do they use this service (per year)?; and Why do they use the service?
If 'No please note this and move to the next service in the list and continue

Physiotherapy

Occupational therapy

Speech therapy

Wellbeing therapy (e.g. counselling)

Hydrotherapy

Wheelchair/buggy services

Continence services

Prenatal diagnosis services

Other (please specify)

28. What social services has your child accessed for alpha-mannosidosis?

Note to interviewer, for each service start by asking whether their child has accessed it. If 'Yes' please ask: At what age did they start using the service (years)?; How often do they use this service (per year)?; and Why do they use the service?
If 'No please note this and move to the next service in the list and continue.

Housing	
Benefits advice	
Direct payment	
Respite short breaks	
Educational healthcare plans	
Carer's assessment	
Other (please specify)	

Alpha-Mannosidosis Disease Progression Survey - II - Carer

For carers of patients with alpha-mannosidosis

Your home

29. Has your home required adaptations to accommodate any disability caused by your child's alpha-mannosidosis? If so, please describe how? What age was your child (years) when the adaptations were made to your home?

30. How were adaptations funded?

- ◯ I funded them myself/my family funded them
- ◯ NHS/Social care funding
- ◯ Council disability facilities grant
- ◯ Housing association grant
- ◯ Part external funding and part personal funding

Alpha-Mannosidosis Disease Progression Survey - II - Carer

For carers of patients with alpha-mannosidosis

Expenses

31. Have you incurred any personal/out of pocket expenses caused by your child's alpha-mannosidosis? If so please estimate the cost per year, at what age (years) and what was the reason for the expense.

Patient Access to Clinical Trials and Treatment

MPS Commercial

Alpha-Mannosidosis Disease Progression Survey - II - Carer

For carers of patients with alpha-mannosidosis

32. Quality of life questionnaires to be administered by interviewer. Carer to respond on behalf of the child. Please tick when completed.

- ◯ HUI-3
- ◯ EQ-5D-5L / EQ-ED-Y

Note to interviewer: Complete EQ-5D-5L for patients aged 16 years and older.

Complete EQ-5D-5Y for patients 8–15 years inclusive; completion by family member, carer of legal guardian

**Legal Guardian: Person with legal authority to care for an individual under the age of 16 years, or an individual 16 years and above without mental capacity to provide informed consent.*

Please record here which version used.

Patient Access to Clinical Trials and Treatment

MPS Commercial

Alpha-Mannosidosis Disease Progression Survey - II - Carer

For carers of patients with alpha-mannosidosis

33. Quality of life questionnaires to be administered by interviewer. Carer's response recorded. Please tick when completed.

- ◯ Caregiver Strain Index
- ◯ Hospital Anxiety and Depression Questionnaire
- ◯ EQ-5D-5L

Patient Access to Clinical Trials and Treatment

MPS Commercial

Alpha-Mannosidosis Disease Progression Survey - III - Patient

For patients with alpha-mannosidosis aged 16 years and older

Overview

1. Patient ID (for office use only)

2. Date of completion

Date: DD / MM / YYYY

3. How old are you?

- ◯ 12–17 years
- ◯ 18 years or older

4. How much do you weigh (kg)?

5. How old were you when you were diagnosed with alpha-mannosidosis (years)?

6. What is your current walking ability? Please tick which best applies.

- ◯ I can walk unaided
- ◯ I can walk with aids (e.g. braces, splints, ankle foot orthoses, crutches, cane, walker)
- ◯ I use a wheelchair
- ◯ I am immobile, need a carer or hoist to transfer, and help from a carer with all daily activities

7. What treatment have you received for alpha-mannosidosis? Please tick all that apply.

- ◯ Supportive/palliative care only
- ◯ Bone marrow transplant
- ◯ Velmanase alfa
- ◯ Other (please specify)

8. How old were you when you started treatment (years)?

Supportive/palliative care only	
Bone marrow transplant	
Velmanase alfa	
Other (please specify)	

Alpha-Mannosidosis Disease Progression Survey - III - Patient

For patients with alpha-mannosidosis aged 16 years and older

Impact of alpha-mannosidosis on health

9. Please describe the impact of alpha-mannosidosis on your health

10. If you have received treatment for alpha-mannosidosis has it impacted on your health? If so, please describe

Alpha-Mannosidosis Disease Progression Survey - III - Patient

For patients with alpha-mannosidosis aged 16 years and older

Impact of alpha-mannosidosis on health-related quality of life (the perception of physical and mental health, e.g. energy and mood)

11. Please describe the impact of alpha-mannosidosis on your health-related quality of life (e.g. how do you feel alpha-mannosidosis affects your physical and mental health?).

12. If you have received treatment for alpha-mannosidosis has it impacted on your health-related quality of life? If so, please describe

Patient Access to Clinical Trials and Treatment

MPS Commercial

Education

13. What type of school did/ do you attend?

- ◯ Mainstream school ± additional support (please describe)
- ◯ Special needs school ± additional support (please describe)
- ◯ Residential school
- ◯ Other (please specify)

14. Were there any changes to your schooling following treatment for alpha-mannosidosis?

- ◯ Not applicable - no treatment received
- ◯ No
- ◯ Yes, please describe

Patient Access to Clinical Trials and Treatment

MPS Commercial

Alpha-Mannosidosis Disease Progression Survey - III - Patient

For patients with alpha-mannosidosis aged 16 years and older

Employment

15. Are you in:

- ◯ Full time work - paid
- ◯ Part time work - paid
- ◯ Full time work - unpaid
- ◯ Part time work - unpaid
- ◯ Unable to work due to alpha-mannosidosis
- ◯ Not applicable to me (e.g. too young to work/in full time education)

16. Has your employment status changed with treatment for alpha-mannosidosis

- ◯ Not applicable - no treatment received
- ◯ No
- ◯ Yes, please describe

Alpha-Mannosidosis Disease Progression Survey - III - Patient

For patients with alpha-mannosidosis aged 16 years and older

Support received

17. Have you received any financial support (e.g. state benefits, grant, one-off payment) resulting from alpha-mannosidosis?

◯ No - Please go to Question 19

◯ Yes, please describe and go to Question 18

18. If you have received treatment for alpha-mannosidosis. Has your financial support changed since you received treatment?

- ○ No - please go to Question 19
- ○ Yes, please describe and go to Question 19

19. Have you received any social care support (e.g. additional carers, respite breaks) resulting from alpha-mannosidosis?

- ○ No - Please go to Question 21
- ○ Yes, please describe and go to Question 20

20. If you have received treatment for alpha-mannosidosis. Has your social care support changed since you received treatment?

- ◯ No - Please go to Question 21
- ◯ Yes, please describe and go to Question 21

Social integration

21. Please describe the impact of alpha-mannosidosis on your social integration (e.g. your motivation and/or ability to meet and communicate with friends, family, or colleagues).

22. If you have received treatment for alpha-mannosidosis, has it impacted on your social integration? If so, please describe:

Patient Access to Clinical Trials and Treatment

MPS Commercial

Alpha-Mannosidosis Disease Progression Survey - III - Carer

For carers of patients with alpha-mannosidosis

Overview

1. Patient ID (for office use only)

[ ]

2. Date of completion

Date DD [ ] / MM [ ] / YYYY [ ]

3. What is your relationship to the patient with alpha-mannosidosis (tick all that apply)?

- ◯ I am a carer
- ◯ I am a family member

4. How old is the patient with alpha-mannosidosis?

- ◯ 6–11 years
- ◯ 12–17 years
- ◯ Aged 18 years and older

5. How much does the patient with alpha-mannosidosis weigh (kg)?

[ ]

6. How old was the patient when they were diagnosed with alpha-mannosidosis (years)?

[ ]

7. What is the patient's current walking ability? Please tick which best applies.

- ◯ They can walk unaided
- ◯ They can walk with aids (e.g. braces, splints, ankle foot orthoses, crutches, cane, walker)
- ◯ They use a wheelchair
- ◯ They are immobile, need a carer or hoist to transfer, and help from a carer with all daily activities

8. What treatment has the patient received for alpha-mannosidosis? Please tick all that apply.

- ◯ Supportive/palliative care only
- ◯ Bone marrow transplant
- ◯ Velmanase alfa
- ◯ Other (please specify)

[ ]

9. How old was the patient when they started treatment (years)?

Supportive/palliative care [ ]

Bone marrow transplant [ ]

Velmanase alfa [ ]

Other (please specify) [ ]

Alpha-Mannosidosis Disease Progression Survey - III - Carer

For carers of patients with alpha-mannosidosis

Impact of alpha-mannosidosis on health

10. Please describe the impact of alpha-mannosidosis on the patient's health.

11. Please describe the impact of alpha-mannosidosis on the carer's health.

12. Please describe the impact of alpha-mannosidosis on the family's health.

13. If the patient has received treatment for alpha-mannosidosis, has it impacted on the health of any of the following. If so, please describe:

The patient

Their carer

Their family

Alpha-Mannosidosis Disease Progression Survey - III - Carer

For carers of patients with alpha-mannosidosis

Impact of alpha-mannosidosis on health-related quality of life (the perception of physical and mental health, e.g. energy and mood)

14. Please describe the impact of alpha-mannosidosis on the patient's health-related quality of life (e.g. how they feel alpha-mannosidosis affects their physical and mental health).

15. Please describe the impact of alpha-mannosidosis on the carer's health-related quality of life (e.g. how they feel the patient's alpha-mannosidosis affects their physical and mental health).

16. Please describe the impact of alpha-mannosidosis on the family's health-related quality of life (e.g. how they feel the patient's alpha-mannosidosis affects their physical and mental health).

17. If the patient has received treatment for alpha-mannosidosis, has it impacted on the health-related quality of life of any of the following. If so, please describe:

The patient

Their carer

Their family

Patient Access to Clinical Trials and Treatment

MPS Commercial

Alpha-Mannosidosis Disease Progression Survey - III - Carer

For carers of patients with alpha-mannosidosis

Education

18. What type of school did/does the patient with alpha-mannosidosis attend?

- ◯ Mainstream school ± additional support (please describe)
- ◯ Special needs school ± additional support (please describe)
- ◯ Residential school
- ◯ Other (please specify)

19. Have there been or were there any changes to the patient's schooling since the patient received treatment for alpha-mannosidosis?

- ◯ No
- ◯ Yes, please describe

Alpha-Mannosidosis Disease Progression Survey - III - Carer

For carers of patients with alpha-mannosidosis

Employment

20. Is the patient in:

- ◯ Full time work - paid
- ◯ Part time work - paid
- ◯ Full time work - unpaid
- ◯ Part time work - unpaid
- ◯ Unable to work due to alpha-mannosidosis
- ◯ Not applicable to this patient (e.g. too young to work/in full time education)

21. Has the patient's employment status changed with their treatment for alpha-mannosidosis

- ◯ No
- ◯ Yes, please describe

22. Is the carer in:

- ◯ Full time work - paid
- ◯ Part time work - paid
- ◯ Full time work - unpaid
- ◯ Part time work - unpaid
- ◯ Unable to work due to caring commitments for a child with alpha-mannosidosis

23. Has the carer's employment status changed with the patient's treatment for alpha-mannosidosis?

- ◯ No
- ◯ Yes, please describe

Alpha-Mannosidosis Disease Progression Survey - III - Carer

For carers of patients with alpha-mannosidosis

Support received

24. Has the patient received any financial support (e.g. state benefits, grant, one-off payment) resulting from alpha-mannosidosis?

- ○ No - Please go to Question 26
- ○ Yes - Please describe and go to Question 25

25. If the patient has received treatment for alpha-mannosidosis. Has their financial support changed since they received treatment?

- No - please go to Question 26
- Yes - please describe and go to Question 26

26. Has the carer received any financial support (e.g. state benefits, grant, one-off payment) resulting from the patient's alpha-mannosidosis?

- No - Please go to Question 28
- Yes - Please describe and go to Question 27

27. If the patient has received treatment for alpha-mannosidosis. Has the carer's financial support changed since the patient received treatment?

- ◯ No - Please go to Question 28
- ◯ Yes - please describe and go to Question 28

28. Has the patient received any social care support (e.g. additional carers, respite breaks) resulting from alpha-mannosidosis?

- ◯ No - Please go to Question 30
- ◯ Yes - Please describe and go to Question 29

29. If the patient has received treatment for alpha-mannosidosis. Has their social care support changed since they received treatment?

- No - Please go to Question 30
- Yes - Please describe and go to Question 30

30. Has the carer received any social care support (e.g. additional carers, respite breaks) resulting from the patient's alpha-mannosidosis?

- No - Please go to Question 32
- Yes - Please please describe and go to Question 31

31. If the patient has received treatment for alpha-mannosidosis. Has the carer's social care support changed since the patient received treatment?

- ◯ No - Please go to Question 32
- ◯ Yes - Please describe and go to Question 32

Alpha-Mannosidosis Disease Progression Survey - III - Carer

For carers of patients with alpha-mannosidosis

Social integration

32. Please describe the impact of alpha-mannosidosis on the patient's social integration (e.g. their motivation and/or ability to meet and communicate with friends, family, or colleagues).

33. Please describe the impact of alpha-mannosidosis on the carer's social integration (e.g. their motivation and/or ability to meet and communicate with friends, family, or colleagues).

34. Please describe the impact of alpha-mannosidosis on the family's social integration (e.g. their motivation and/or ability to meet and communicate with friends, family, or colleagues).

35. If the patient has received treatment for alpha-mannosidosis, has it impacted on the social integration of any of the following. If so, please describe:

The patient

Their carer

Their family
